# High level of correspondence across different news domain quality rating sets

**DOI:** 10.1093/pnasnexus/pgad286

**Published:** 2023-09-02

**Authors:** Hause Lin, Jana Lasser, Stephan Lewandowsky, Rocky Cole, Andrew Gully, David G Rand, Gordon Pennycook

**Affiliations:** Hill/Levene Schools of Business, University of Regina, 3737 Wascana Parkway, Regina, SK, S4S 0A2, Canada; Sloan School of Management, Massachusetts Institute of Technology, 100 Main St, Cambridge, MA 02142, USA; Department of Psychology, Cornell University, Uris Hall, 211, Tower Rd, Ithaca, NY 14853, USA; Institute for Interactive Systems and Data Science, Graz University of Technology, Inffeldgasse 16C, 8010 Graz, Austria; Complexity Science Hub Vienna, Josefstädterstraße 39, 1080 Vienna, Austria; School of Psychological Science, University of Bristol, 12a, Priory Road, Bristol BS8 1TU, UK; School of Psychology, University of Western Australia, 35 Stirling Hwy, Crawley, WA 6009, Australia; Jigsaw (Google LLC), 1600 Amphitheatre Parkway, Mountain View, CA 94043, USA; Jigsaw (Google LLC), 1600 Amphitheatre Parkway, Mountain View, CA 94043, USA; Sloan School of Management, Massachusetts Institute of Technology, 100 Main St, Cambridge, MA 02142, USA; Department of Brain and Cognitive Sciences, Massachusetts Institute of Technology, 43 Vassar St, Cambridge, MA 02139, USA; Hill/Levene Schools of Business, University of Regina, 3737 Wascana Parkway, Regina, SK, S4S 0A2, Canada; Department of Psychology, Cornell University, Uris Hall, 211, Tower Rd, Ithaca, NY 14853, USA; Department of Psychology, University of Regina, 3737 Wascana Parkway, Regina, SK S4S 0A2, Canada

**Keywords:** misinformation, fact-checking, news quality, journalism standards

## Abstract

One widely used approach for quantifying misinformation consumption and sharing is to evaluate the quality of the news domains that a user interacts with. However, different media organizations and fact-checkers have produced different sets of news domain quality ratings, raising questions about the reliability of these ratings. In this study, we compared six sets of expert ratings and found that they generally correlated highly with one another. We then created a comprehensive set of domain ratings for use by the research community (github.com/hauselin/domain-quality-ratings), leveraging an ensemble “wisdom of experts” approach. To do so, we performed imputation together with principal component analysis to generate a set of aggregate ratings. The resulting rating set comprises 11,520 domains—the most extensive coverage to date—and correlates well with other rating sets that have more limited coverage. Together, these results suggest that experts generally agree on the relative quality of news domains, and the aggregate ratings that we generate offer a powerful research tool for evaluating the quality of news consumed or shared and the efficacy of misinformation interventions.

**Significance Statement**

Researchers, policymakers, and technology companies often quantify how much misinformation people share or are exposed to by looking at the quality of the news domains that they interact with. It remains unclear, however, to what extent different experts agree on the relative quality of different domains. This study compared ratings from different media organizations and fact-checkers and found that they correlated well with one another. We then used a “wisdom of experts” approach to aggregate these ratings to create the most comprehensive set of 11,520 domain ratings to date. Our results indicate that experts generally agree on the relative quality of news domains and our new set of ratings with extensive coverage offers a tool for evaluating content quality.

## Introduction

Misinformation is not new, but concern over the problem has grown in recent years among researchers, policymakers, and technology companies. A large body of research has focused on understanding why people consume or share misinformation (1–4), and many interventions have been developed to combat the spread of misinformation online (5–7). Despite growing interest in the problem, most research has skirted around fundamental measurement problems that are crucial to scientific progress and policy development (8, 9). Here, we focus on the problem of assessing news domain quality. We compare different quality measures, evaluate the correspondence between these measures, and propose an aggregate measure of domain quality.

A common measurement challenge in studies of misinformation is quantifying information quality. Many studies have taken the approach of classifying posts flagged by third-party fact-checkers as misinformation (6, 8, 10–12). Fact-checkers, however, are able to evaluate only a small fraction of the content posted online every day, and therefore, this approach is not scalable.

To address the scalability problem when analyzing social media data, researchers from many fields have used evaluations of the quality of news sites or domains as a whole, instead of the individual pieces of content found on these sites (2, 3, 13–18). This domain-level approach is based on the premise that journalistic standards differ systematically across news domains, such that higher-quality domains (e.g. Reuters) generally produce higher-quality content than lower-quality domains (e.g. Infowars). However, different media organizations and fact-checkers evaluate domains along different, and sometimes nonoverlapping, dimensions (e.g. bias, factuality, and transparency), raising questions about the degree of agreement across different expert ratings. To the extent that different rating systems disagree, this creates a substantial measurement problem when using domain-level quality ratings.

We address this measurement problem by first showing that different expert ratings generally agree on the relative quality of domains. We then leveraged a “wisdom of expert crowds” or “ensemble model” approach to create a more comprehensive aggregate rating set that researchers can use (https://github.com/hauselin/domain-quality-ratings). Our rating set includes 11,520 domains, spanning a comprehensive range from high quality (e.g. Reuters and Associated Press) to low quality (e.g. Natural News and Before It's News). Given the current lack of consensus across researchers on which rating set to use, and the limited coverage of some widely used rating sets, our aggregate set of ratings addresses important measurement problems in the field, which will result in better tools for quantifying misinformation, evaluating interventions, and facilitating theory development (9).

## Results

**Fig. 1.**
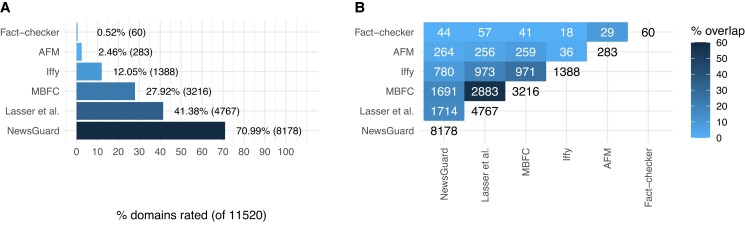
A) Percentage and number of domains (of 11,520) with ratings for 6 expert sources. B) Overlaps in rated domains between sources. The diagonal white cells indicate the total number domains with ratings for a given source. The off-diagonal elements indicate, for a given pair of sources, the number of domains that have been rated by both sources. Percent overlap and shading reflect the intersection of domains rated by a given pair of sources divided by the union of domains rated by the same pair of sources. Professional fact-checkers from Pennycook and Rand (16), AFM, Iffy index of unreliable sources (Iffy), MBFC, Lasser et al. (19), and NewsGuard.

We obtained quality ratings for 11,520 domains from 6 diverse expert sources that included media organizations and independent professional fact-checkers (Fig. 1). These six expert sources used different scoring systems and evaluated domains on different dimensions (e.g. trust, reliability, (un)biasedness, credibility, and transparency). Our analyses are the first to directly examine whether expert sources that evaluate domains using different scoring systems and criteria produce domain ratings that correspond with one another (see Materials and Methods for more information on each source).

The number of domains with ratings varied across the six sources (Fig. 1A), which included many nonoverlapping domains (Fig. 1B). For example, 60 domains have been rated by independent professional fact-checkers (16), 4,767 by Lasser et al. (19), and 8,178 by NewsGuard (as of 2022 September 13). Of the domains rated by NewsGuard, 44 have also been rated by fact-checkers (60 rated domains), and 264 rated by Ad Fontes Media (AFM; 283 rated domains), and 1,691 rated by Media Bias/Fact-Check (MBFC; 3,216 rated domains). Table 1 shows 12 example domain ratings for the 6 sources. Twelve of the 11,520 domains are not typically considered as news sites (youtube.com, google.com, facebook.com, apple.com, blogspot.com, medium.com, wordpress.com, msn.com, yahoo.com, gettr.com, go.com, and radio.com), and they are excluded from subsequent analyses.

**Table 1.** Example domain ratings.

Domain	NewsGuard	MBFC	AFM	Fact-checker	Lasser	Iffy binary
nytimes	1.00	0.83	0.81	1.00	0.88	1.00
washingtonpost	–	0.75	0.81	1.00	0.88	1.00
foxnews	0.57	0.58	0.61	0.48	0.50	0.00
huffingtonpost	–	0.83		0.52	0.50	1.00
chicagotribune	0.92	0.83	0.90	0.58	0.88	1.00
usatoday	–	0.92	0.89	0.73	0.88	1.00
breitbart	0.50	0.50	0.51	0.18	0.00	0.00
whatdoesitmean	–	0.11		0.00	0.00	1.00
beforeitsnews	0.00	0.42	0.01	0.00	0.00	0.00
newsmax	–	0.50	0.49	0.14	0.00	0.00
commondreams	–	0.75	0.60	0.03	0.38	1.00
rawstory	–	0.58	0.53	0.10	0.38	1.00

Ratings are normalized, such that a value of 0 indicates lowest quality, and a value of 1 indicates highest quality. Several NewsGuard ratings have been redacted (–) because NewsGuard permits the publication of only five example ratings. Empty cells are domains that have not been rated by a given source. Lasser, Lasser et al. (19) mean. Iffy binary: Since the Iffy index includes only unreliable domains (*n* = 1,388), the Iffy binary measure was created by assigning all domains included in the Iffy source a 0 and assigning all the domains not included in the Iffy source a 1.

### Original nonimputed ratings distributions and correlations

**Fig. 2.**
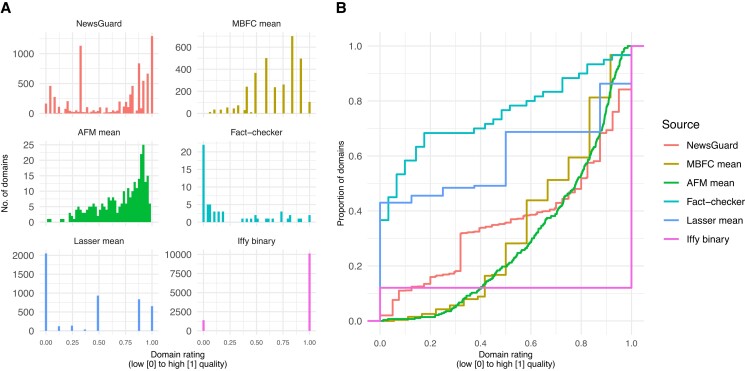
A) Distributions of ratings where each panel is one source. B) Empirical cumulative distribution functions, *P*(rating ≤ *x*)—the proportion of domains that is equal to or less than a given rating *x*. Iffy, Iffy index of unreliable sources; Lasser, Lasser et al. (19) mean.

Figure 2A shows the different distributions of domain ratings for the six sources. For example, the 60 ratings by professional fact-checkers from Pennycook and Rand (16) have a strong right skew (i.e. most included domains are of low quality), whereas AFM's 283 ratings are left-skewed (most included domains are of high quality). These differences in distributions highlight a critical measurement issue that researchers face when defining and quantifying misinformation using domain ratings: The prevalence of misinformation is likely to vary considerably depending on the set of ratings used and the quality threshold used to define a domain as low quality (20). According to NewsGuard's guideline, a domain with a NewsGuard rating below 0.60 “generally fails to meet basic standards of credibility and transparency” (21). Many studies have followed NewsGuard's guideline (18, 19), but because the different rating scales are not commensurate, if one were to apply the same 0.60 threshold to a different set of ratings, different domains would be considered as containing misinformation. To illustrate this point, the empirical cumulative distributions in Fig. 2B show the proportion of domains that is equal to or less than a given threshold or rating, separately for each source. For example, 80% of the domains rated by fact-checkers are scored below 0.60, but only 29% of AFM's domains are below 0.60.

Despite the different distributions, however, the correlations in Fig. 4A suggest substantial agreement across the six sources in the relative quality of the various domains (Pearson correlation coefficient *r* range: 0.32 to 0.86; Fig. 4A, top triangle). Since the distributions are non-normal, we also report nonparametric Spearman rank correlations (*ρ* range: 0.32 to 0.90; Fig. 4A, bottom triangle), which also suggest substantial agreement across sources. Three of the six sources (MBFC, AFM, and Lasser) rated domains on more than one dimension (e.g. reliability and unbiasedness), and the distributions of ratings of these additional dimensions also vary considerably (Fig. S1), but they correlated relatively strongly with one another (Fig. S2).

### Combining ratings via imputation and principal component analysis

The relatively strong correlations observed in Fig. 4A suggest that there is general agreement on the relative ranking of domains across the six sources (even though they use different scoring systems and criteria), and that domain quality might be a latent variable that could be recovered by aggregating the data. To do so, we combine imputation (see Materials and Methods for details) with principal component analysis (PCA): Imputation is necessary because there are missing values (i.e. there are different numbers of rated domains per source; Fig. 1) and is suitable given the correlations across the sources; a PCA performed on the imputed data can produce a component that serves as an aggregate rating. Note that because 3 of the 6 sources rated domains on multiple dimensions (MBFC, AFM, and Lasser; Figs. S1 and S2; see Materials and Methods for details), we have 16 sets of ratings from these 6 sources, and the imputation and PCA were performed on all 16 sets of ratings (imputed data and PC1 can be found here: https://github.com/hauselin/domain-quality-ratings).

**Fig. 3.**
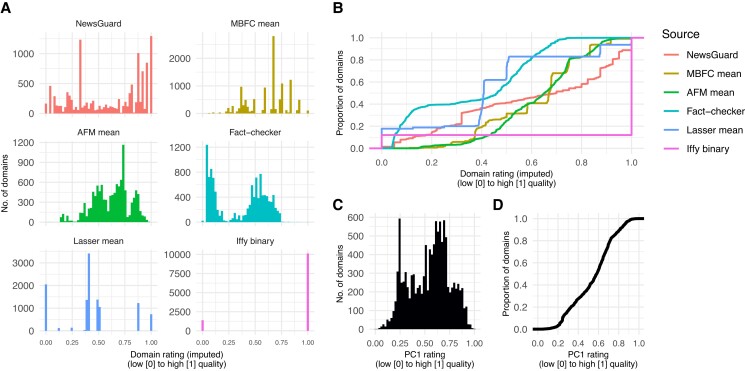
A) Distributions of imputed ratings where each panel is one source. B) Empirical cumulative distribution functions, *P*(rating ≤ *x*)—the proportion of domains that is equal to or less than a given rating *x*. Distribution of PC1 ratings. C). Distribution of PC1 ratings. D). Empirical cumulative distribution function for PC1 ratings.

The distributions of the imputed ratings for the various sources (Fig. 3A and B) retain some of the characteristics of the original, nonimputed distributions (Fig. 2A and B). For example, the imputed fact-checker and AFM ratings retain some of their right and left skew, respectively (compare Figs. 2A and 3A). Table 2 shows the imputed ratings for select domains (sorted by PC1), and Figs. S3 and S4 show the distributions and correlations of imputed ratings for the 16 sets of ratings.

**Table 2.** Example imputed domain ratings sorted by PC1.

Domain	PC1	NewsGuard	MBFC	AFM	Fact-checker	Lasser	Iffy
reuters	1.00	–	1.00	0.96	0.67	1.00	1.00
afp	0.95	–	0.92	0.95	0.67	1.00	1.00
economist	0.93	–	0.92	0.87	0.66	1.00	1.00
usatoday	0.90	–	0.92	0.89	0.73	0.88	1.00
chicagotribune	0.87	0.92	0.83	0.90	0.58	0.88	1.00
nytimes	0.86	1.00	0.83	0.81	1.00	0.88	1.00
washingtonpost	0.82	–	0.75	0.81	1.00	0.88	1.00
theguardian	0.75	–	0.67	0.78	0.77	0.88	1.00
huffingtonpost	0.72	–	0.83	0.73	0.52	0.50	1.00
commondreams	0.54	–	0.75	0.60	0.03	0.38	1.00
foxnews	0.53	0.57	0.58	0.61	0.48	0.50	0.00
rawstory	0.46	–	0.58	0.53	0.10	0.38	1.00
breitbart	0.30	0.50	0.50	0.51	0.18	0.00	0.00
newsmax	0.29	–	0.50	0.49	0.14	0.00	0.00
zenith.news	0.23	–	0.42	0.47	0.05	0.00	0.00
healthnutnews	0.15	–	0.06	0.48	0.08	0.00	0.00
whatdoesitmean	0.15	–	0.11	0.37	0.00	0.00	1.00
beforeitsnews	0.06	0.00	0.42	0.01	0.00	0.00	0.00
infowars	0.05	–	0.11	0.16	0.03	0.00	0.00
naturalnews	0.00	–	0.11	0.03	0.07	0.00	0.00

A value of 0 indicates lowest quality, and a value of 1 indicates highest quality. Several NewsGuard ratings have been redacted (–) because NewsGuard permits the publication of only five example ratings. Iffy, Iffy index of unreliable sources; Lasser, Lasser et al. (19) mean.

The first principal component (PC1) of the PCA performed on the imputed data set explains 68.21% of the variance in the data, and the distribution of PC1 ratings resembles the aggregate of the other sources (Fig. 3C and D). Note that this aggregate PC1 rating does not include NewsGuard ratings, which were removed to satisfy NewsGuard's requirements to not publish aggregates that include their ratings. Nonetheless, the results are very similar when including NewsGuard's ratings, and Fig. S6 suggests the aggregate PC1 ratings are relatively robust to variations in the number of sources included in the PCA.

**Fig. 4.**
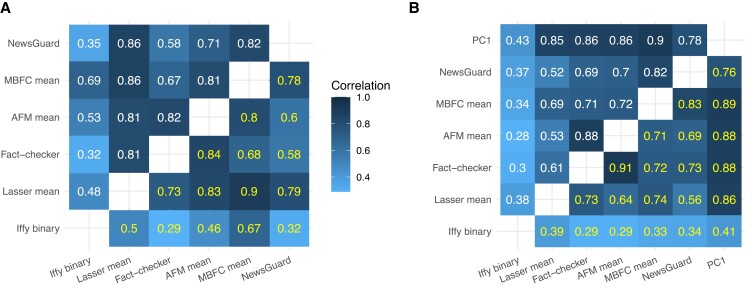
A) Raw (nonimputed) domain rating correlations. B) Imputed domain rating correlations. Values in the upper triangle are Pearson's *r* and values in the lower triangle are Spearman's *ρ*. Iffy, Iffy index of unreliable sources; Lasser, Lasser et al. (19) mean.

**Fig. 5.**
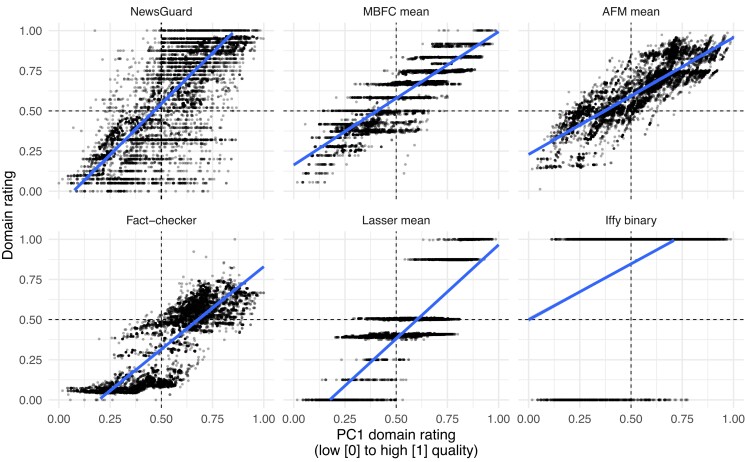
Scatterplots showing the relationships between the PC1 and other imputed source ratings. Each dot is one domain. Iffy, Iffy index of unreliable sources; Lasser, Lasser et al. (19) mean.

The PC1 ratings span a wide range from low (0) to high (1) quality (see Table 2): Examples of lower-quality domains include Natural News (0.00), Gateway Pundit (0.11), and Daily Kos (0.41); examples of medium-quality domains include Daily Caller (0.47) and MSNBC (0.59); and those of higher-quality domains include Reuters (1.00), Chicago Tribune (0.87), and New York Times (0.86). Importantly, the PC1 ratings correlate highly with the other imputed ratings (Figs. 4B and 5; min *r* = 0.43, or min *r* = 0.67, if Iffy binary is excluded; see Fig. S5 for PC1 correlations with all 16 dimensions). These results suggest that PC1 could serve as an aggregate rating because it captures a substantial amount of signal that is common to the different sources.

Since many ratings across the different sources have been imputed, the correlations across sources are generally expected to be lower than the correlations in the original, nonimputed data set. Moreover, for sources that assigned noncontinuous ratings (Iffy binary or ordinal Lasser ratings), they correlated slightly worse with other sources after imputation, partly because of the increased number of tied ratings.

### Robustness checks and validations

We perform robustness checks and validations on our data. Domains vary in terms of their importance or popularity, and we examine whether the correlations that we observe in the imputed data are robust after accounting for domain importance, which we quantified using the Open PageRank metric (22). It uses open-source data to recreate Google's PageRank scores, which Google stopped sharing publicly since 2016. Out of 11,520 domains, 8,776 are in Open PageRank's top 10 million domains, suggesting that the majority of our domains are relatively important. When we weight correlations using Open PageRank's scores (Fig. S7), the resulting correlations are almost identical to the unweighted correlations shown in Figs. 4B and S4. In addition, to validate our combined imputation-PCA approach, we perform simulations to show that the imputation strategy recovers the simulated missing data and PC1 ratings relatively well (Fig. S8).

## Discussion

We find that the domain-quality ratings provided by six different expert sources correlate highly with one another. Despite using different criteria to evaluate domains, different experts produce similar relative domain rankings. This finding indicates that there are common perceptions of relative differences in journalistic standards, which suggests that these differences in domain ratings may be veridical.

By showing agreement across different expert ratings for the first time, our results provide evidence for the utility of fact-checked domain ratings for evaluating content quality. Prior studies have found less agreement, because they either defined agreement as the number of overlapping domains between different fake news lists (20) or included less credible and nonexpert ratings (23). Our work not only addresses these limitations, but also combines imputation with dimension reduction to produce a large new set of aggregate ratings that address previous problems (e.g. which fake news list one should use). Importantly, the comprehensive set of continuous ratings that we provide could encourage more nuanced and realistic questions like “what is the quality of a domain” than binary questions like “is this domain a fake news producer or not.”

Of pragmatic significance, the list of 11,520 domains in our aggregate set is, to our knowledge, the most comprehensive list of domain ratings to date. The PC1 explained almost 70% of the variance in the data, and the PC1 ratings correlated highly with the other imputed ratings (except for the Iffy index of unreliable sources). Thus, our PC1 ratings can serve as an aggregate domain quality measure, harnessing the “wisdom of experts” to produce an ensemble rating. The benefit of this approach is that biases unique to any particular set of ratings are less likely to have an influence, and the resulting ratings cover far more domains than any of the other existing rating lists. Naturally, if biases that are present across all of the sources for domain ratings, then they will nonetheless manifest in our imputed scores.

In addition to facilitating research, the aggregate ratings could also be used as part of interventions against misinformation. For example, algorithms can focus more on lower-quality domains that are important or popular (e.g. inverse of PC1 weighted by domain importance), such that content from these domains is more likely to be flagged for human fact-checking. Recent work shows that displaying quality labels on social media posts can discourage the sharing of false claims (24), and our aggregate ratings could be used to label domains at scale. In addition, researchers can regularly perform our imputation and aggregation procedure to recompute PC1 to identify trends that suggest that a domain may be becoming a fake news producer (e.g. decreasing PC1 ratings over time). Finally, topic modeling of content from domains with different PC1 ratings could provide insights into the different forms of misinformation. For example, domains with PC1 ratings lower than 0.10 might be producing clearly fake news, whereas those with ratings between 0.20 and 0.40 might contain more false advertisements, or unchecked and misleading claims that might not necessarily be false (25).

A limitation of using domain ratings is that it assumes each domain produces content of identical quality, which is rarely (and perhaps never) the case. For example, the New York Times has a relatively high PC1 rating of 0.86, but its content can sometimes be fairly left-leaning or biased, even though factually accurate (26). Conversely, lower-quality domains often reproduce content from other higher-quality domains (such as the Associated Press), and therefore, their content is certainly not always inaccurate. However, this issue is inherent in any domain-level rating scheme. Although recruiting professional fact-checkers to check each individual piece of content would help avoid this issue, doing so is extremely time-consuming and expensive. Thus, domain-level ratings offer an approach that trades off some precision in order to gain tractability.

## Conclusion

In summary, our results suggest that there is substantial agreement across different sources of domain quality ratings, and that aggregated domain ratings provide a useful tool for advancing misinformation research. Domain ratings may not be as accurate as fact-checking individual pieces of content, but they offer a convenient tool for evaluating the efficacy of antimisinformation interventions. When used alongside other approaches like misinformation detection algorithms, professional fact-checkers, digital literacy education, and crowdsourced fact-checking, domain ratings can be a valuable tool for combating and studying misinformation.

## Materials and methods

We obtained domain quality ratings from six different sources that included media organizations and professional fact-checkers. The raw, nonimputed data (excluding NewsGuard data that are proprietary) and code can be found at https://doi.org/10.17605/osf.io/9jwzs. These sources rated overlapping sets of domains (Fig. 1B), and we included them because they used different scoring systems and evaluation criteria, which allow us to evaluate whether experts—despite using different evaluation approaches—produce domain ratings that correspond with one another. To standardize the scores before further processing, we separately normalized each set of ratings such that the continuous domain ratings ranged from 0 (lowest quality) to 1 (highest quality).

### Sources

#### NewsGuard

NewsGuard is a journalism and technology organization that rates the credibility of news and information websites. It evaluates domains on nine apolitical criteria, which are weighted differently to create a “Nutrition Label” (27). A rating of 100 indicates high credibility (“website adheres to all nine standards of credibility and transparency”) and 0 indicates “website is unreliable because it severely violates basic journalistic standards.” As of November 2022, NewsGuard's proprietary database included 8,178 domains. Because NewsGuard recommends to “proceed with caution” for domains with ratings below 60, researchers have generally dichotomized the scores using the threshold 60 (18, 19, 28), which also used to be the official recommendation of NewsGuard (21).

#### Media Bias/Fact-Check

Media Bias/Fact-Check (https://mediabiasfactcheck.com) is an independent website found by Dave Van Zandt and maintained by a team of researchers and journalists. It relies on human fact-checkers affiliated with the International Fact-Checking Network to evaluate media sources along different dimensions such as factual reporting and bias (29). We managed to obtain ratings for 3,216 sources (as of November 2022). MBFC rates domains on two relevant dimensions: factualness (“MBFC accuracy”) and biasedness (which we recoded as unbiasedness: “MBFC unbias”). We created two additional composite ratings from the “MBFC accuracy” and “MBFC unbias” dimensions by computing their mean (“MBFC mean”) and minimum (“MBFC min”).

#### Ad Fontes Media

Ad Fontes Media (https://adfontesmedia.com) is a public benefit corporation that rates news articles on two dimensions: reliability (“AFM reliability”) and biasedness (which we recoded as unbiasedness: “AFM unbias”). Each article is rated by at least three human analysts with balanced left, right, and center self-reported political viewpoints (for methodology, see Ad Fontes Media [30]). We managed to obtain 283 freely available rated domains (as of November 2022) from their website. We created two additional composite ratings from the “AFM reliability” and “AFM unbias” dimensions by computing their mean (“AFM mean”) and minimum (“AFM min”).

#### Independent professional fact-checkers

Sixty domain ratings were obtained from Pennycook and Rand (16), who recruited eight professional fact-checkers from the Poynter International Fact-Check Network. The fact-checkers rated 60 domains based on how much they trusted each one. This set of domains contains 20 mainstream media outlets (e.g. npr.org), 22 websites that produce hyperpartisan coverage of actual facts (e.g. dailykos.com), and 18 websites that produce fake news (e.g. now8news.com). They were selected from lists generated by different sources such as BuzzFeed News, PolitiFact, and Grinberg et al. (2), and other websites (for details, see Pennycook and Rand [16]).

#### Lasser et al. (2022)

Lasser et al. (19) compiled lists of problematic sites curated by different media and fact-checking organizations (e.g. Fake news watch, Columbia Journalism review, and Décodex), and devised a coding system to combine the ratings across 4,767 unique domains (see GitHub repository README for lists and methodology: https://github.com/JanaLasser/misinformation_domains). They rated domains on accuracy, reliability, and transparency. We computed the mean (“Lasser mean”) and minimum (“Lasser minimum”) ratings using the accuracy and transparency dimensions (the third dimension reliability was excluded because it was already computed using the other two dimensions).

#### Iffy index of unreliable sources

The Iffy index (https://iffy.news) is one of the most extensive lists of unreliable or fake news domains (1,388 domains as of November 2022). It includes domains that have been assigned a low credibility rating by MBFC (which has evaluated thousands of unreliable and reliable domains; see next section). Note that it does not incorporate MBFC's political leaning ratings and has been used in many peer-reviewed studies (31). Since this index includes only unreliable or low credibility domains, all domains included in this index were assigned 0, whereas the remaining 10,132 unrated domains in our list were assigned 1, and we call this set of ratings “Iffy binary.”

### Imputation

We perform multiple imputations with the Python library miceforest to fill in the missing values. The algorithm used gradient-boosted trees (LightGBM) (32) to impute missing data with an iterative method known as Multiple Imputation by Chained Equations (33, 34). We tune the hyperparameters of the model and generate multiple imputed data sets with different parameter combinations. Note that only missing values were imputed—that is, in the imputed data sets, nonmissing values retained their original values. For each imputed data set, we compute pairwise correlations between the imputed ratings by different sources, and we select the imputed data set with the highest overall mean correlation. The imputed ratings can be found here (NewsGuard ratings are omitted to satisfy NewsGuard's requirements regarding not publishing their data): https://github.com/hauselin/domain-quality-ratings.

## Supplementary Material

pgad286_Supplementary_Data

## Data Availability

Full reproduction materials, including data and analysis code, but excluding the NewsGuard proprietary database, are accessible at https://doi.org/10.17605/osf.io/9jwzs. To acquire data from NewsGuard, contact support@newsguardtech.com.
