# Supplementary material for: High level of correspondence across different news domain quality rating sets
Source: PNAS Nexus. 2023 Sep 2;2(9):pgad286. doi: 10.1093/pnasnexus/pgad286 (PMC10500312; doi:10.1093/pnasnexus/pgad286)
Supplement: pgad286_Supplementary_Data [file pgad286_supplementary_data.docx]

# Supplementary Information for

High level of correspondence across different news domain quality rating sets


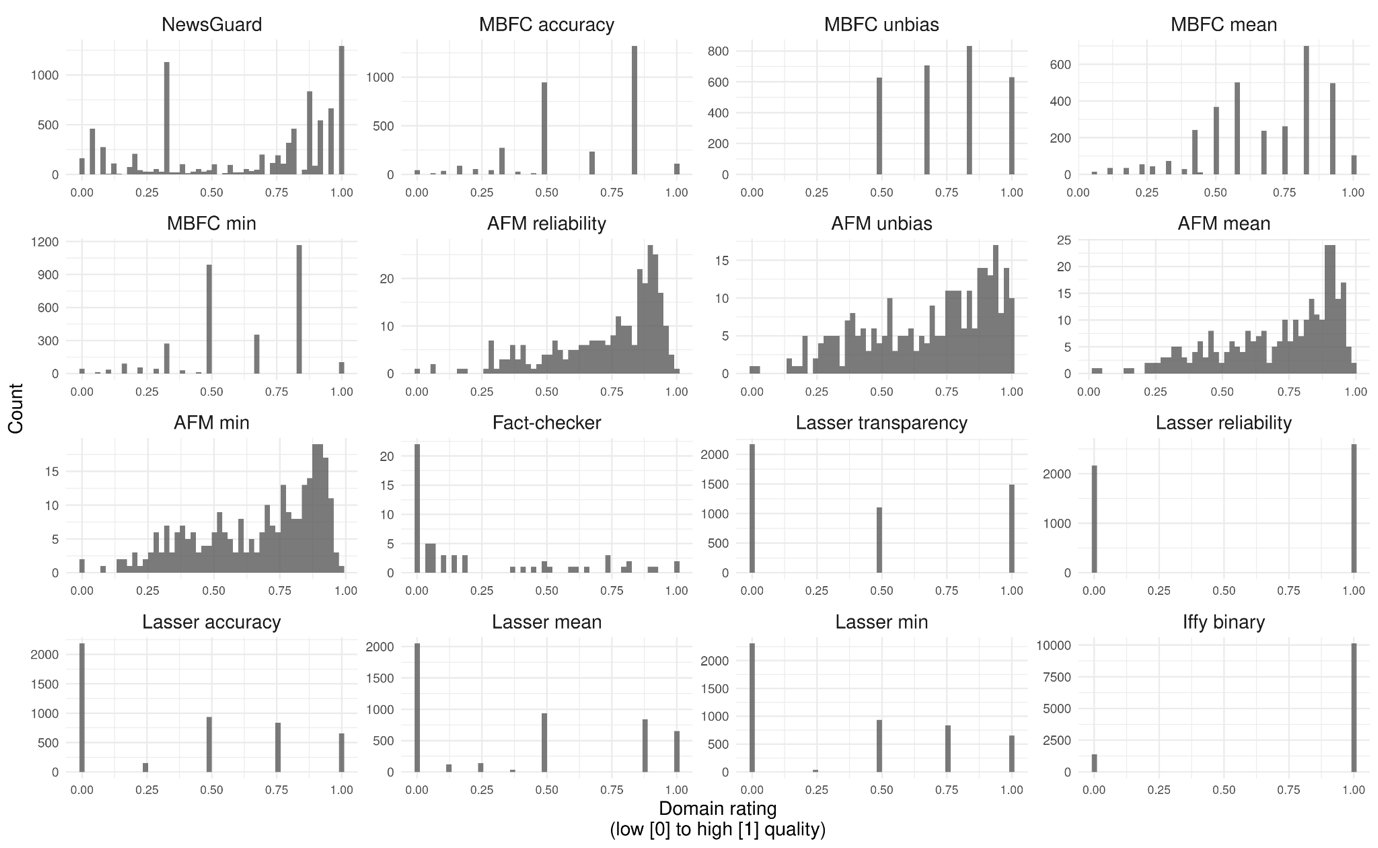


**Figure S1.** Distributions of ratings for each source. Each panel is one source. The quality ratings on the x-axis range from 0 (lowest quality) to 1 (highest quality). MBFC: Media Bias/Fact Check. AFM: Ad Fontes Media. Lasser: (1). Iffy: Iffy index of unreliable sources.

**
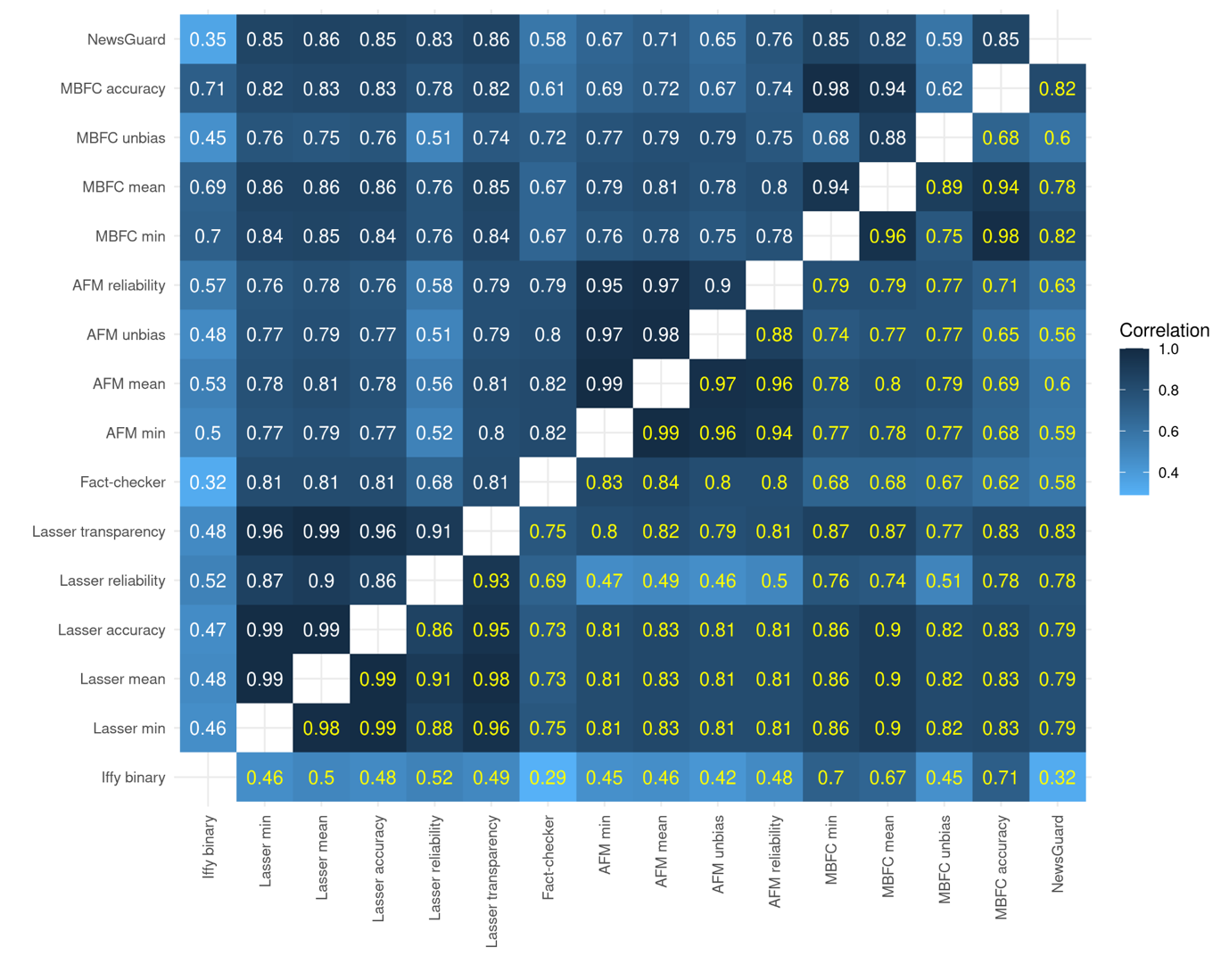
**

**Figure S2.** Domain rating correlations. Values in the upper triangle are Pearson’s *r* and values in the lower triangle are Spearman’s *ρ*. MBFC: Media Bias/Fact Check. AFM: Ad Fontes Media. Lasser: (1). Iffy: Iffy index of unreliable sources.


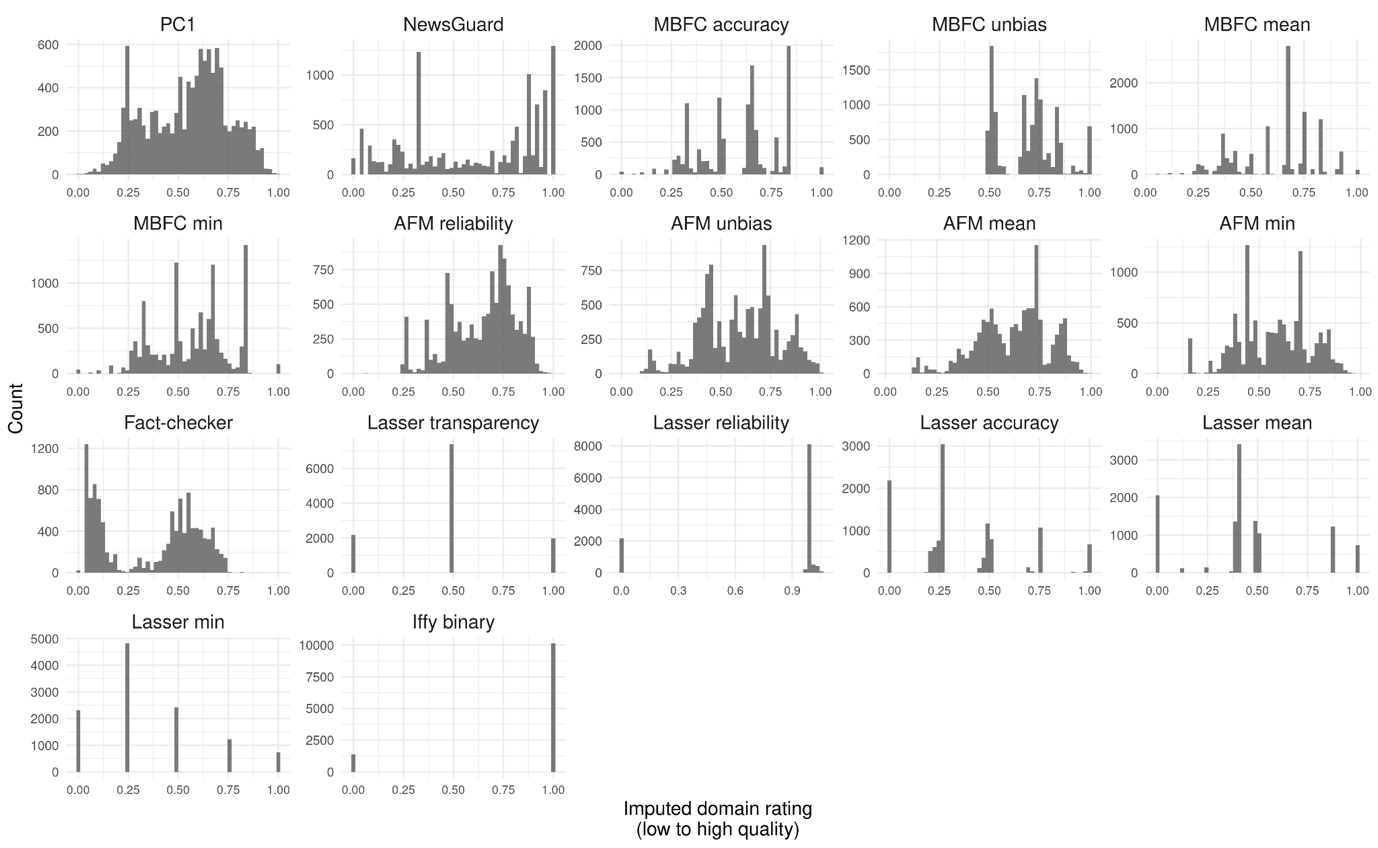


**Figure S3**. Distributions of imputed ratings for each source. PC1: first principal component. MBFC: Media Bias/Fact Check. AFM: Ad Fontes Media. Lasser: (1). Iffy: Iffy index of unreliable sources.


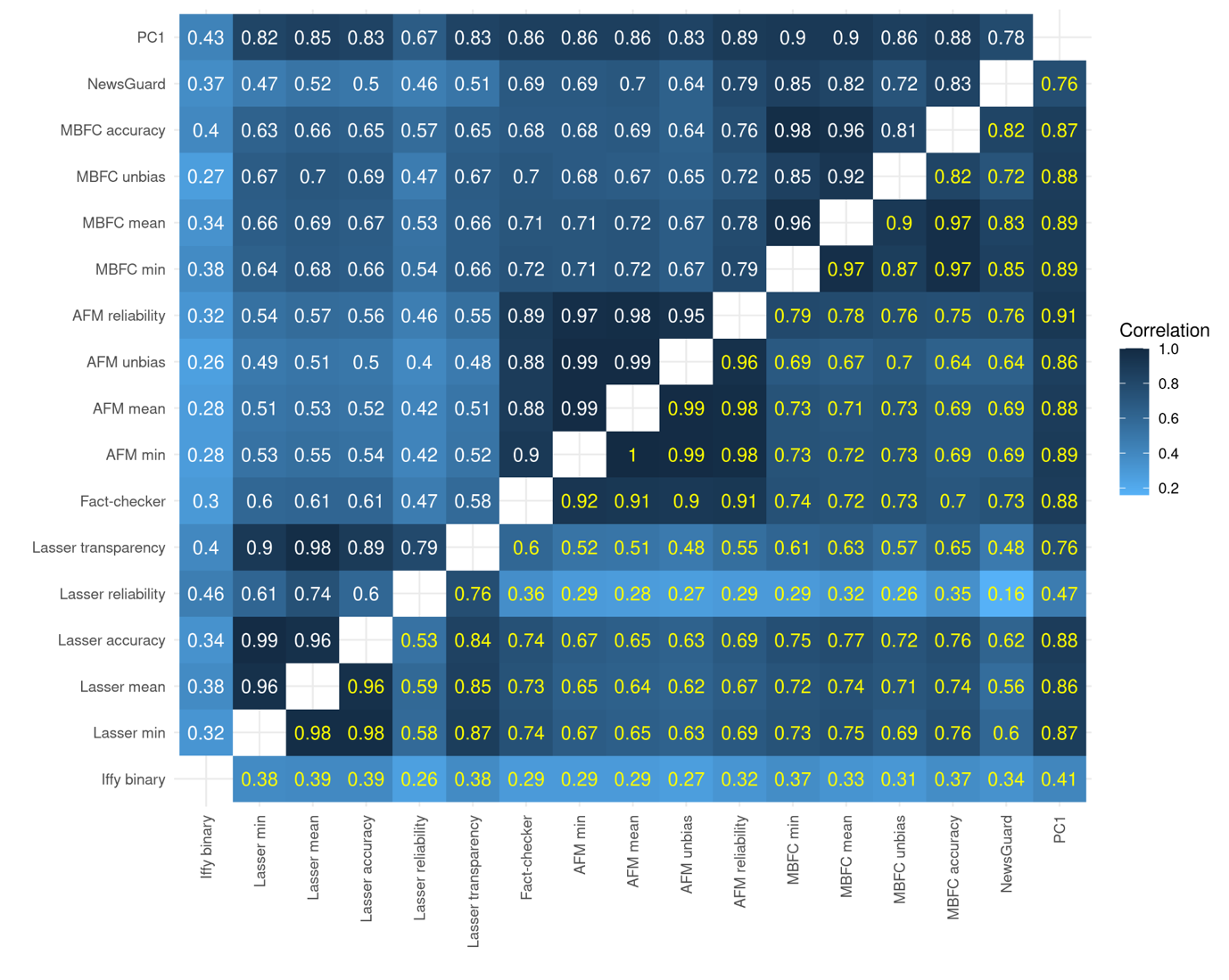


**Figure S4**. Imputed domain rating correlations. Values in the upper triangle are Pearson’s *r* and values in the lower triangle are Spearman’s *ρ*. PC1: first principal component. MBFC: Media Bias/Fact Check. AFM: Ad Fontes Media. Lasser: (1). Iffy: Iffy index of unreliable sources.


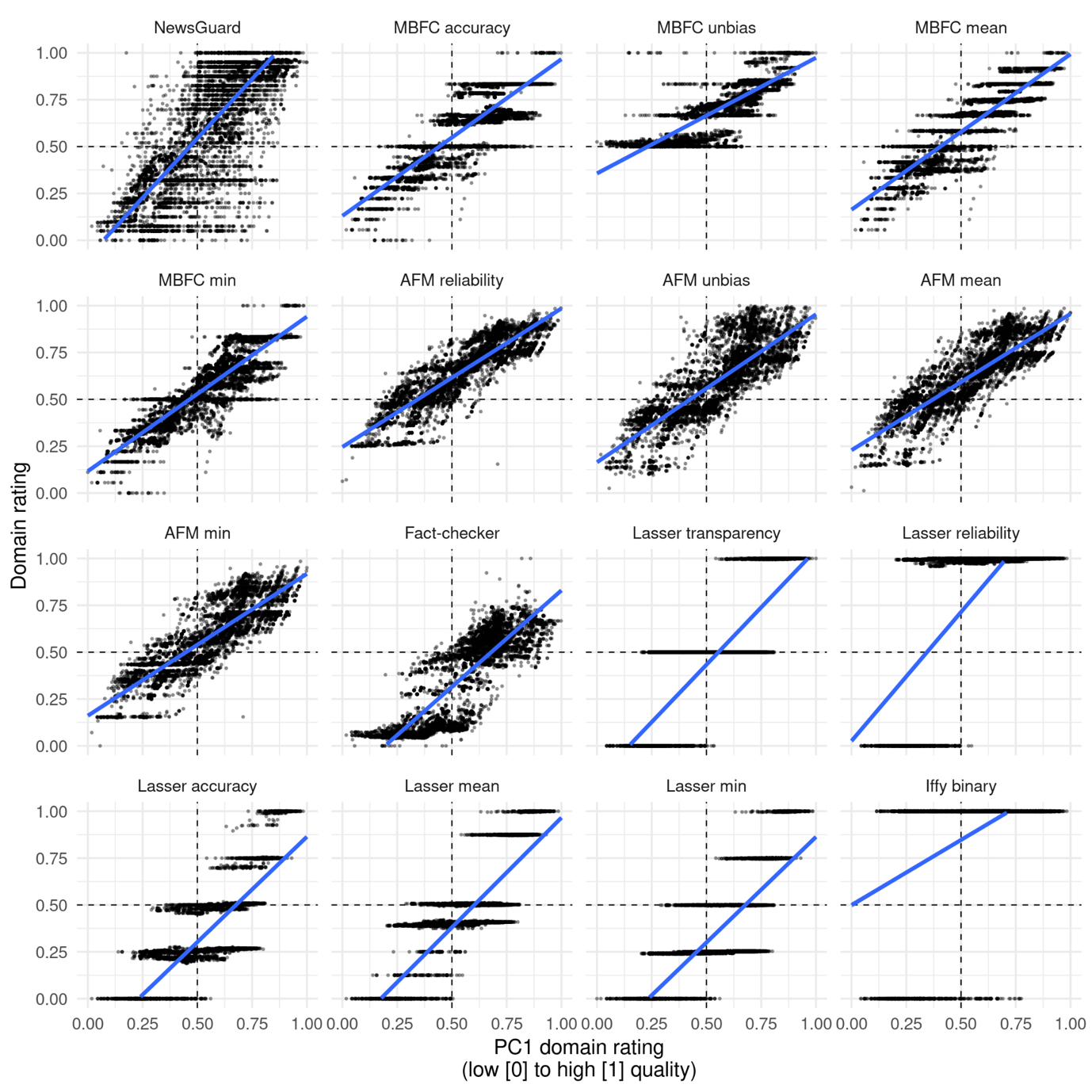


**Figure S5.** Scatterplots showing the relationships between the first principal component (PC1) and other imputed source ratings. Each dot is one domain.PC1: first principal component. MBFC: Media Bias/Fact Check. AFM: Ad Fontes Media. Lasser: (1). Iffy: Iffy index of unreliable sources.


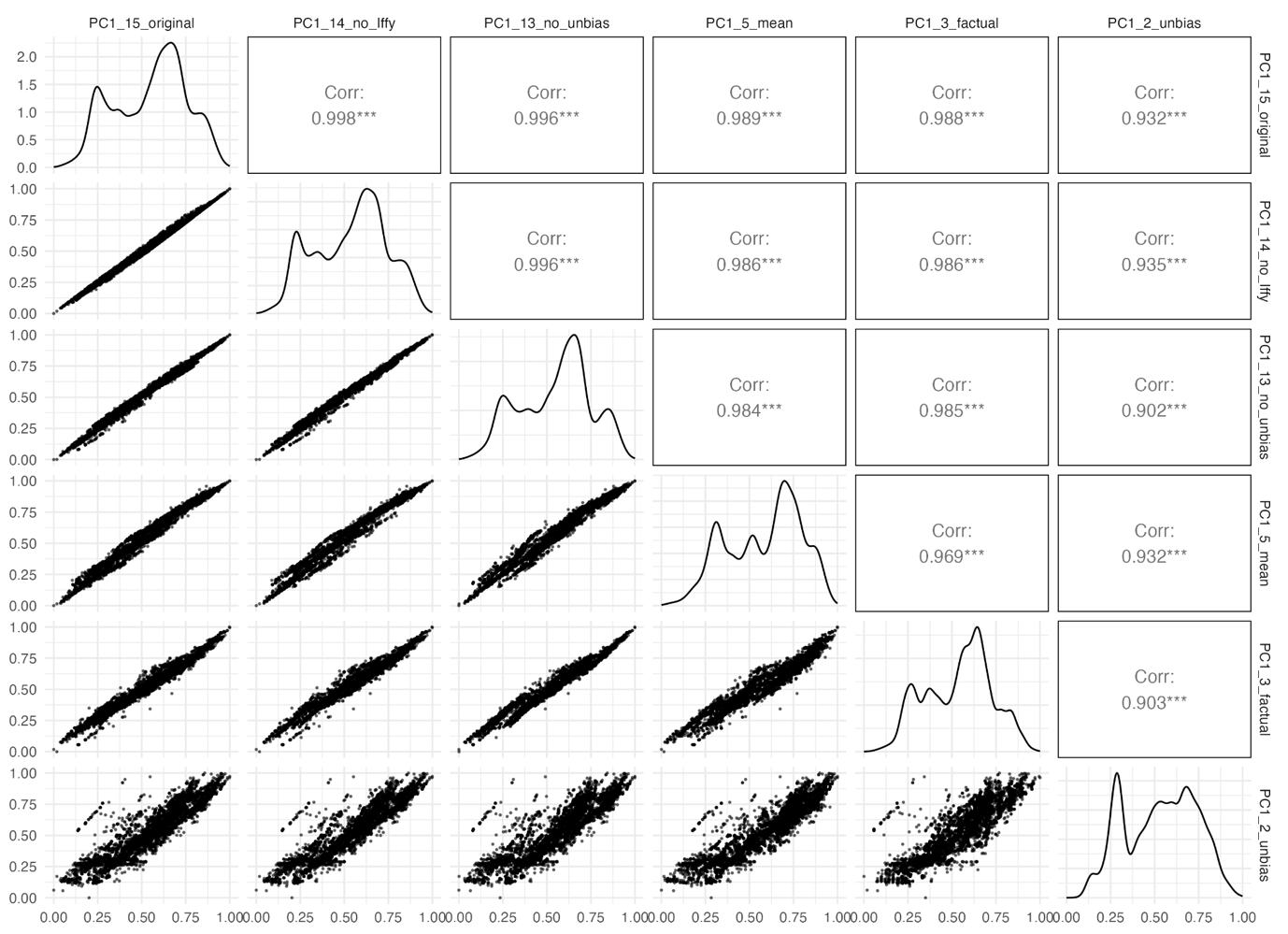


## Figure S6. PC1 correlations for six PCAs that used different input vectors. The first column and row (PC1_15_original) is the PC1 vector reported in the main text. PC1_14_no_Iffy has 14 input vectors (excluding Iffy binary); PC1_13_no_unbias has 13 input vectors (excluding AFM unbias and MBFC unbias); PC1_5_mean has 5 input vectors (Fact-checker, AFM mean, MBFC mean, Lasser mean, Iffy binary); PC1_3_factual has 3 input vectors (AFM reliability, MBFC accuracy, Lasser accuracy); PC1_2_unbias has 2 input vectors (AFM unbias, MBFC unbias).

##

##
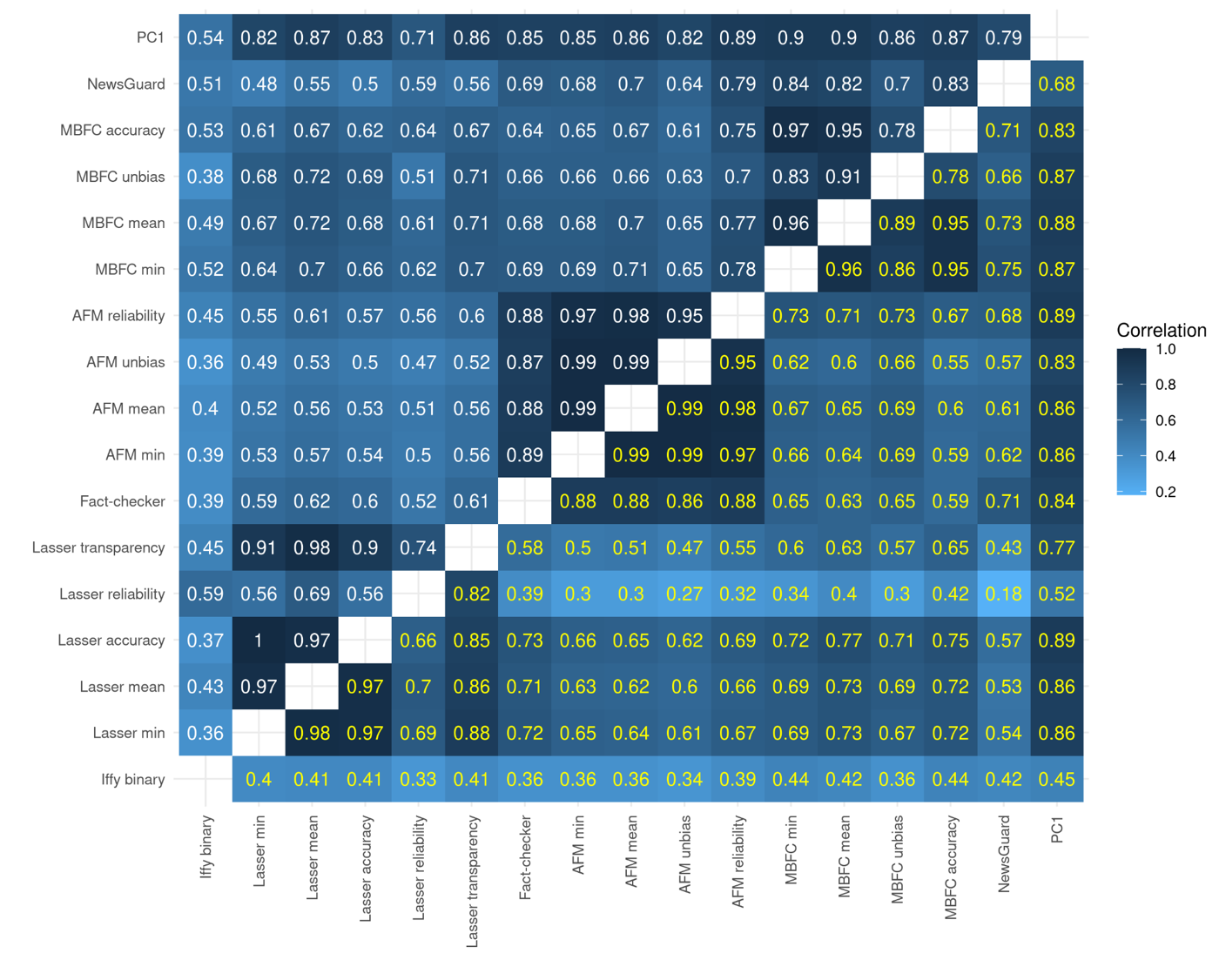
 Figure S7. Imputed domain rating weighted correlations. Correlations are weighted by Open PageRank’s domain importance metric. Values in the upper triangle are Pearson’s *r* and values in the lower triangle are Spearman’s *ρ*. PC1: first principal component. MBFC: Media Bias/Fact Check. AFM: Ad Fontes Media. Lasser: (1). Iffy: Iffy index of unreliable sources.

##

To validate the imputation method, we simulate data by drawing samples from a multivariate normal distribution to create a simulated dataset that has the same correlations as our imputed data (the number of samples is the number of domains and the covariance of this multivariate distribution mirrors the observed imputed domain correlations shown in Figure 5).

First, we perform PCA on this simulated dataset (without any missing values) and find that PC1 explains 67.95% of the variance in the data. Next, we introduce missing values such that the total number of missing values in each variable/source was identical to the original data, but were *missing randomly*. We perform imputation and PCA on this data, and find that PC1 explains 67.84% of the variance. Finally, we introduce missing values in the simulated dataset to recreate the non-random missing value structure in the original dataset. We then perform imputation and PCA, and find that PC1 explains 63.32% of the variance. Supplementary Figure 8 shows the two PC1s from the two imputed datasets correlated highly with the PC1 from the simulated data without missing values (left and middle panels). The PC1 from the dataset with data missing non-randomly (middle panel) corresponds better with the PC1 from the data without missing values (*r* = 0.73 and the correlation/regression line is closer to the diagonal), relative to PC1 from the dataset with data missing at random (left panel; *r* = 0.64). These simulations indicate that the imputation method works relatively well for our missing value data structure.

**
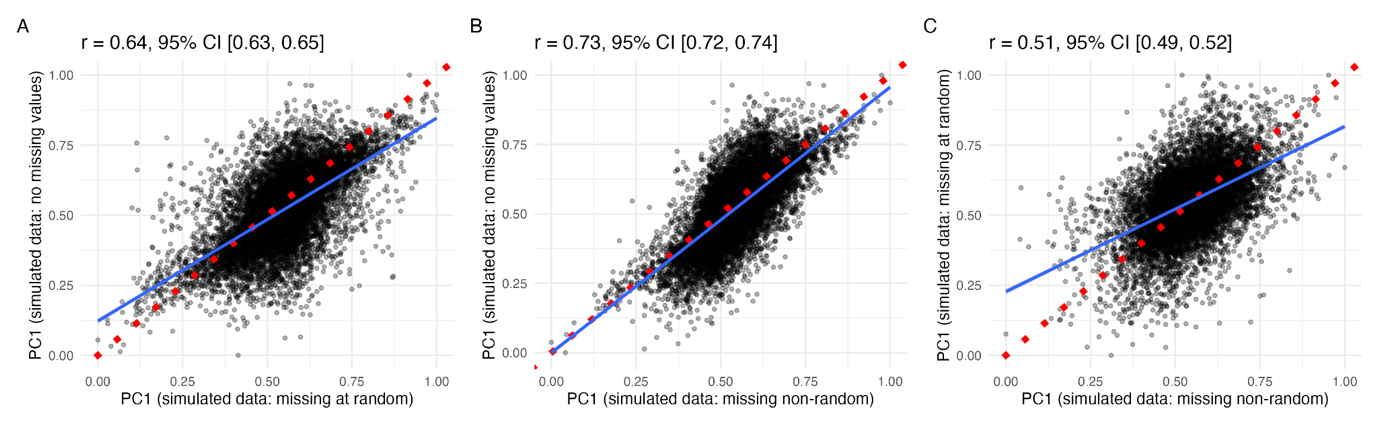
**

**Figure S8**. Correlation between PC1 from the simulated data without missing values and PC1 from the simulated data with data missing at random (**A**). Correlation between PC1 from the simulated data without missing values and PC1 from the simulated data with data missing non-randomly that replicates the missing data structure of the observed data (**B**). Correlation between PC1 from the simulated data with data missing at random and PC1 from the simulated data with data missing non-randomly (**C**). The blue line is the correlation/regression line; the red dotted line is the diagonal.

References

1. Lasser J *et al.* 2022. Social media sharing by political elites: An asymmetric American exceptionalism. *PNAS Nexus*
